# Fertility sparing surgery vs radical surgery for epithelial ovarian cancer: a meta-analysis of overall survival and disease-free survival

**DOI:** 10.1186/s12885-020-06828-y

**Published:** 2020-04-15

**Authors:** Denghua Liu, Jing Cai, Aiwei Gao, Zehua Wang, Liqiong Cai

**Affiliations:** 1 Department of Neurology, Tongji Hospital, Tongji Medical College, Huazhong University of Science and Technology, Wuhan, Hubei 430030 People’s Republic of China; 2 Department of Obstetrics and Gynecology, Union Hospital, Union Medical College, Huazhong University of Science and Technology, Wuhan, Hubei 430022 People’s Republic of China

**Keywords:** Conservative surgery, Ovarian cancer, Fertility, Survival

## Abstract

**Background:**

The aim of this systematic review and meta-analysis was to compare overall survival and disease-free survival after fertility sparing surgery (FSS) vs radical surgery in stage 1 epithelial ovarian cancer (EOC).

**Methods:**

A systematic literature search of PubMed, BioMed Central, Scopus, CENTRAL (Cochrane Central Register of Controlled Trials) and Google scholar was carried out. Databases were searched for English language studies from inception to 1st November 2019. Adjusted hazard ratios (HR) were extracted and pooled for a meta-analysis. Meta-regression was performed for baseline patient characteristics.

**Results:**

Eight observational studies compared 2223 patients undergoing FSS with 5809 patients undergoing radical surgery. Overall survival was reported from all eight studies. The pooled HR was non-significant (HR, 1.03; 95%CI, 0.80–1.31; *p* = 0.84) denoting no difference in overall survival between FSS and radical surgery. Data on disease-free survival was available from five studies. Our analysis indicated no difference in disease-free survival between EOC patients undergoing FSS or radical surgery (HR, 1.07; 95%CI, 0.73–1.58; *p* = 0.72). On meta-regression, there was no a statistically significant effect of cancer stage, grade and histology on the pooled HR.

**Conclusion:**

On the basis of currently available observational studies there seems to be no difference in overall survival and disease-free survival with either surgical techniques for stage 1 EOC patients. Disease stage, tumor grade and histology does not appear to influence outcomes. Further homogenous studies shall improve the quality of evidence on this debatable subject.

## Background

Ovarian cancer is one of the most common malignancy of the female reproductive tract [1]. Epithelial ovarian carcinoma (EOC) consists of a large sub-group of ovarian cancer and is usually seen in post-menopausal women. Despite a predominance in older age, around 10% of EOC are diagnosed in women at the age of 40 years or less [2]. One important dilemma in managing such patients with EOC is preservation of reproductive function. Radical surgery with bilateral oophorectomy, hysterectomy and omentectomy in young patients leads to loss of reproductive potential and menopause thereby resulting in decreased quality of life, grief, distress and sexual dysfunction [3]. With a greater proportion of women delaying childbearing due to lifestyle changes, it is important to discuss the role of fertility-sparing surgery (FSS) with young EOC patients who still wish to conceive.

FSS consists of unilateral salpingo-oophorectomy, pelvic and para-aortic lymphadenectomy, peritoneal biopsies and omentectomy. The contralateral ovary and the uterus are left in situ to preserve fertility [4]. However, it is also important to preserve the survival rate of cancer patients undergoing FSS. There has been a general consensus that FSS may be offered to patients with borderline, germ cell and stromal ovarian tumors [5]. A number of observational studies have also demonstrated that FSS in stage 1 EOC may not decrease survival rates and it may be carried out in patients desirous of preserving fertility [5, 6].

In the absence of randomized controlled trials, data from retrospective studies has been the only available evidence to clinicians involved in the management of EOC patients. Evidence from case-control studies is however plagued by small sample size and presence of confounding factors. While multivariate regression analysis has been utilized by many studies to adjust for baseline characteristics for reporting outcomes, no attempt has been made till date to collate such data and analyze the overall evidence on FSS vs radical surgery for stage 1 EOC patients. We hereby present the results of the first meta-analysis of adjusted hazard ratios (HR) for overall survival and disease-free survival comparing FSS and radical surgery for the management of stage 1 EOC.

## Methods

### Criteria for study inclusion

The review was performed following the PRISMA statement (Preferred Reporting Items for Systematic Reviews and Meta-analyses) [7] and the Cochrane Handbook for Systematic Reviews of Intervention [8]. Following the PICOS (Population, Intervention, Comparison, Outcome, and Study design) outline, we included any peer-reviewed study carried out on a *Population* of adult patients with ovarian cancer comparing FSS (*Intervention)* with radical surgery (*Comparison*) and reporting overall survival and/or disease-free survival as an *outcome* variable. There was no restriction placed on the histological type and grade of epithelial ovarian cancer. Studies focussing solely on advanced (Stage 2 or 3) EOC and studies > 10% of FSS patients with stage 2 EOC were excluded. Additionally, we excluded: 1. Studies with < 20 patients with FSS 2. Studies on borderline ovarian tumours 3. Studies with mean follow-up of < 2 years 4. Studies not reporting hazard ratios (HR) of overall survival and disease-free survival 5. Studies not reporting separate baseline data of patients undergoing FSS and radical surgery 6. Case series, case reports, letter to editors and abstracts. In case of studies with overlapping data, the study reporting the largest dataset was included.

### Search strategy

A systematic literature search of various electronic databases including PubMed, BioMed Central, Scopus, CENTRAL (Cochrane Central Register of Controlled Trials) and Google scholar was carried out. Databases were searched for English language studies from inception to 1st November 2019. Two independent reviewers performed the literature search using the MeSH terms and free-text keywords. “Fertility sparing surgery”, “Conservative surgery”, “ovarian cancer”, “epithelial ovarian cancer”, and “epithelial ovarian tumours” were used in various combinations. We manually checked the reference lists of all included studies and review articles for any additional references. The literature search results were screened by their titles and abstracts by two independent reviewers for every database. Potentially relevant articles were then extracted and subsequently screened by their full text. Both the reviewers assessed individual studies based on inclusion criteria and resolved any disagreement, by discussion. The detailed search strategy and results of PubMed database are presented in Supplementary content 1.

### Data extraction and risk of bias assessment

A data abstraction form was used by the reviewers to source data from the selected studies. Details of authors, publication year, inclusion/exclusion criteria, sample size, demographic data, histological type, cancer stage and grade, adjuvant radiotherapy or chemotherapy, study outcomes (adjusted HR for overall survival and disease-free survival) and follow-up were extracted. The outcomes of interest were to assess the difference in overall survival and disease-free survival between patients undergoing FSS and radical surgery.

### Statistical analysis

Adjusted HR for FSS vs radical surgery extracted from the included studies were pooled for a meta-analysis. Study estimates were combined using inverse variance-weighted averages of logarithmic HRs in a fixed-effects model. Heterogeneity was calculated using the I^2^ statistic. I^2^ values of 25–50% represented low, values of 50–75% medium and more than 75% represented substantial heterogeneity. A sensitivity analysis was performed to assess the contribution of each study to the pooled estimate by excluding individual studies one at a time and recalculating the pooled HR estimates for the remaining studies. Publication bias was assessed by visual inspections of funnel plots. The software “Review Manager” (RevMan, version 5.3; Nordic Cochrane Centre [Cochrane Collaboration], Copenhagen, Denmark; 2014) was used for the meta-analysis. To assess the effect of baseline variables on the pooled effect size, a fixed-effects meta-regression analysis was carried out using SPSS statistical software version 23. We assessed the impact of the proportion of patients with specific cancer histology (mucinous, endometroid, serous, clear cell), cancer stage (Stage IA/IB or Stage IC), cancer grade (low grade: grade 1&2, high grade: grade 3) and undergoing adjuvant radio/chemotherapy on the pooled HR. Effect of each moderator is presented as meta-regression coefficient with 95% confidence interval (CI). Meta-regression coefficients demonstrate the estimated increase in logHR per unit increase in the moderator. As logHR = 0 corresponds to HR = 1, 95% confidence intervals crossing the 0 value (i.e. intervals varying from negative to positive values) denote no effect of the moderator on the overall outcome. Coefficients with negative values indicate that as the given factor increases, HR decreases i.e. better survival with FSS.

## Results

**Fig. 1:**
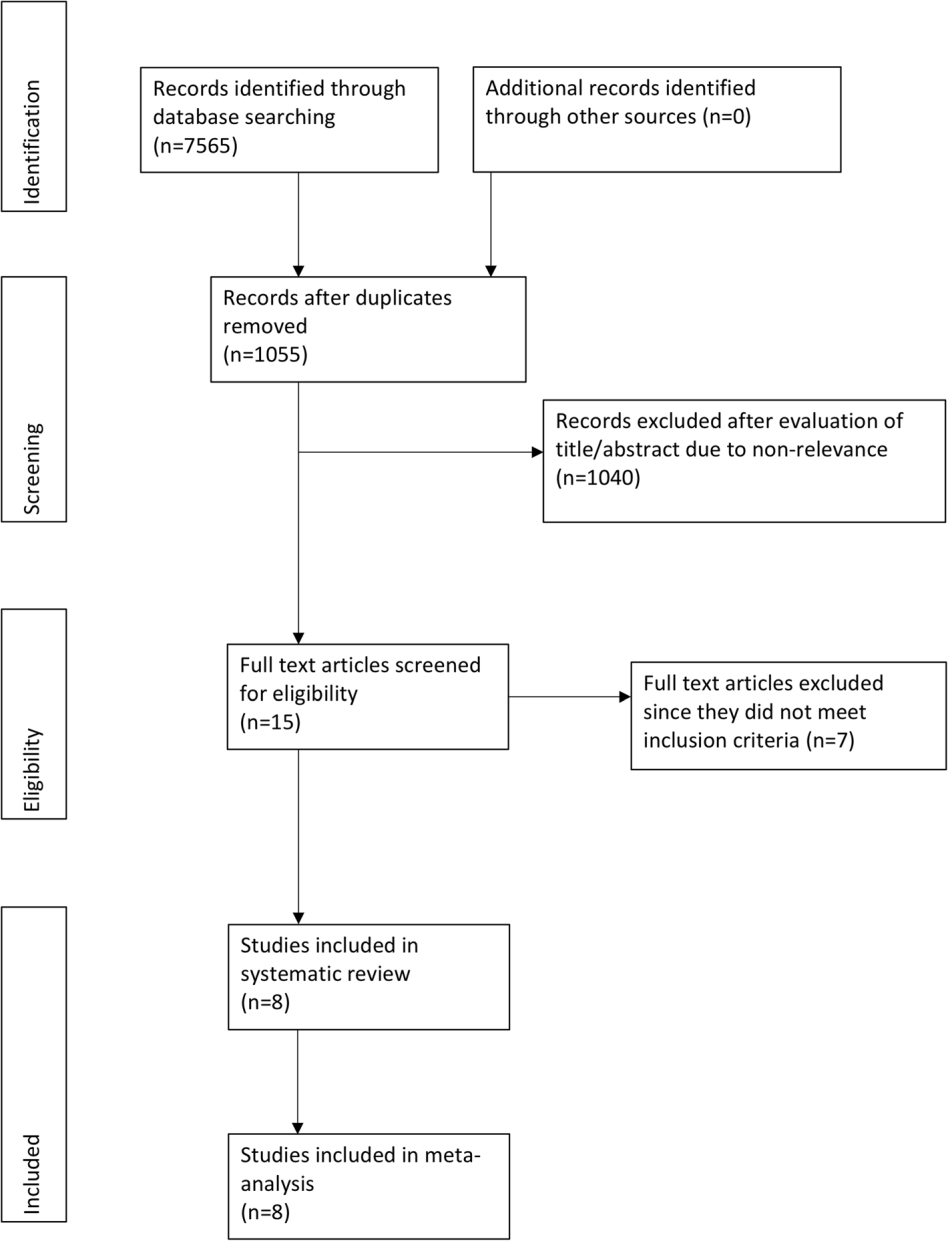
Study flow chart

On an exhaustive literature search, 15 studies were identified for full text review (Fig. 1). Seven studies were excluded as they did not meet the inclusion criteria [9–15]. Four studies were excluded due to overlapping data set [9, 10, 12, 13], two has a sample of less that 20 patients undergoing FSS [11, 14] while one did not report HR [15]. A total of eight studies were included in this systematic review and meta-analysis [5, 6, 16–21]. A total of 2223 patients undergoing FSS were compared with 5809 patients undergoing radical surgery in these studies. One study reported data from two cancer registries which was potential overlapping [21]. Data of the registry with the maximum participants was included. In another study [17], the authors retrospectively analyzed data into two sub-groups: unilateral ovary preservation or uterus preservation. To avoid duplication of data, unilateral oophorectomy data was included in the meta-analysis.

All included studies were retrospective in nature. The number of patients undergoing FSS varied from 35 to 1262. The description of FSS varied amongst studies but all included preservation of unilateral ovary (Table 1). The age of patients undergoing FSS was significantly less as compared to those undergoing radical surgery. One study [19] included patients with mucinous tumors only while all major histopathological types of EOC were included in the remaining studies. With the exception of three studies [6, 19, 21], all studies included patients with stage 1 EOC. Separate HR for stage 1 EOC were available from one study [21] while in the remaining two studies [6, 19] the percentage of stage 2 cases in FSS group were < 10%.

**Table 1:** Characteristics of included studies

Author	Year	Sample size		FSS	RS	Age group (yr)		Histology		Adjuvant chemo/radio therapy		Stage		Grade		Follow-up (yr)
		FSS	RS			FSS	RS	FSS	RS	FSS	RS	FSS	RS	FSS	RS	
Crafton et al	2020	1262	3823	Unilateral salpingo-oophorectomy and uterine preservation	Bilateral salpingo-oophorectomy or hysterectomy	Range: 15–44		Mucinous: 461 Endometroid: 418 Serous: 309 Clear cell: 368	Mucinous: 682 Endometroid: 1308 Serous: 1465 Clear cell: 368	Y:651 N: 611	Y:2569 N: 1254	IA/B: 590 IC: 321 II-IV: 351	IA/B: 1242 IC: 792 II-IV: 1789	1or2:956 3:306	1or2:2339 3:1484	4.6^1^
Jiang et al	2017	52	56	Ipsilateral adnexectomy and biopsy or wedge excision of contralateral ovary	Hysterectomy and bilateral adnexectomy	25^1^	35^1^	Mucinous: 38 Endometroid: 1 Serous: 1 Clear cell: 2	Mucinous: 14 Endometroid: 16 Serous: 8 Clear cell: 18	Y:33 N:19	Y:50 N:6	IA/B: 19 IC1: 24 IC2/3:9	IA/B: 17 IC1: 31 IC2/3:8	1:45 2:4 3:1	1:27 2:8 3:3	6.9^1^
Frusico et al	2016	242	789	Cystectomy or unilateral adnexectomy and complete peritoneal staging	Hysterectomy, bilateral salpingo-oophorectomy	31.3^2^	53.1^2^	Mucinous: 101 Endometroid: 55 Serous: 62 Clear cell: 17 Others:7	Mucinous: 152 Endometroid: 203 Serous: 237 Clear cell: 134 Others:56	Y:103 N:139	Y:494 N:295	IA: 129 IB: 2 IC1: 57 IC2/3:46	IA: 337 IB: 63 IC1: 136 IC2/3:223	1:145 2:69 3:27	1:225 2:225 3:320	11.9^1^
Lee et al	2015	35	55	Unilateral salpingo-oophorectomy with surgical exploration. Surgical staging was optional	Patients other than those undergoing FS surgery	28.6^2^	50.3^2^	Mucinous:35	Mucinous:55	Y:14 N:21	Y:26 N:29	IA: 21 IB: 0 IC: 13 II: 1	IA: 34 IB: 1 IC: 17 II: 3	1:27 2:5 3:1 NK:2	1:34 2:15 3:2 NK:4	8.6^1^
Ditto et al	2015	70	237	Preservation of the uterus, of at least one tube and the contralateral ovary	Radical comprehensive staging	30^1^	51^1^	Mucinous: 36 Endometroid: 8 Serous: 18 Others: 8	Mucinous: 2 Endometroid: 71 Serous: 83 Others: 48	Y:26 N:44	Y:169 N:68	IA: 46 IB: 2 IC: 15 II-III: 6	IA: 95 IB: 21 IC: 45 II-III: 76	1:36 2:24 3:9	1:64 2:80 3:85	FSS:6.4^1^ RS:6.8^1^
Kajiyama et al	2011	74	498	Conservation of the uterus and contralateral ovary and fallopian tube with at least a full peritoneal staging	Hysterectomy, and bilateral salpingo-oophorectomy with peritoneal staging	≤40	< 40 and > 40 age groups	Mucinous: 43 Endometroid: 14 Serous: 4 Clear cell: 13 Others:0	Mucinous: 107 Endometroid: 114 Serous: 60 Clear cell: 199 Others: 18	Y:54 N:20	Y:392 N:106	IA: 36 IB: 1 IC1: 21 IC2/3:16	IA: 155 IB: 6 IC1: 145 IC2/3:192	1or2:57 3:4	1or2:266 3:33	FSS:5.2^1^ RS:5.5^1^
Wright et al	2009	432	754	Unilateral oophorectomy	Bilateral oophorectomy	< 25:666 25–35: 181 36–50:185	< 25:64 25–35: 68 36–50:682	Mucinous: 211 Endometroid: 90 Serous: 98 Clear cell: 33	Mucinous: 188 Endometroid: 285 Serous: 160 Clear cell: 121	Y: 426 N:3 NK:3	Y: 720 N:22 NK:12	1A:370 1C:62	1A:551 1C:203	1:157 2:92 3:37	1:236 2:196 3:104	NS (5-year survival reported)
Zanetta et al	1997	56	43	Unilateral oophorectomy and uterine preservation and surgical staging	Hysterectomy, and bilateral salpingo-oophorectomy with surgical staging	29^2^	34^2^	Mucinous: 23 Endometroid: 13 Serous: 18 Clear cell: 0 Others:2	Mucinous: 10 Endometroid: 10 Serous: 16 Clear cell: 5 Others:02	Y:16 N:40	Y:10 N:33	IA: 32 IB: 2 IC: 22	IA: 27 IB: 3 IC: 13	1:35 2:14 3:7	1:21 2:15 3:7	7.83^1^

^1^Median value; ^2^Mean value

FSS, Fertility sparing surgery; RS, Radical surgery; yr, years; Y, yes; N, No; NS, Not specified; NK, Not known

Due to difference in baseline variables between the FSS and radical surgery groups, we did not pool the number of patients who survived or with recurrence for a meta-analysis. HR ratios adjusted for dependent variables were extracted from the included studies and pooled together for a meta-analysis.

### Outcomes

**Fig. 2:**
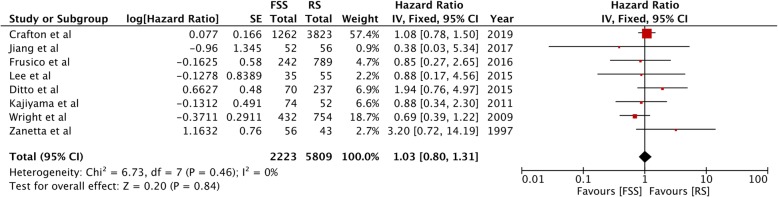
Forrest plot of hazard ratios for overall survival comparing fertility sparing surgery with radical surgery

**Fig. 3:**
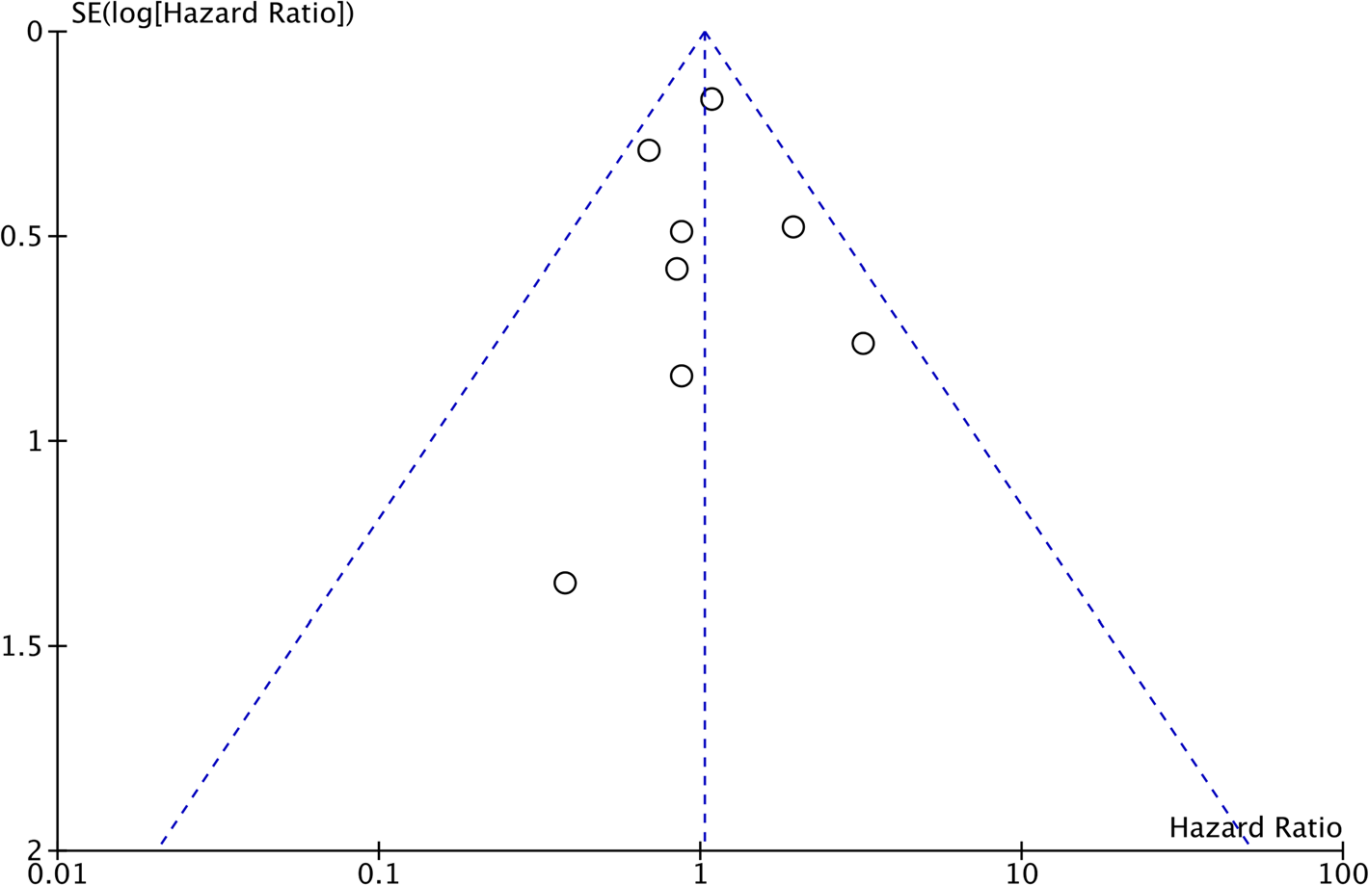
Funnel plot of studies comparing overall survival of fertility sparing surgery with radical surgery

**Fig. 4:**
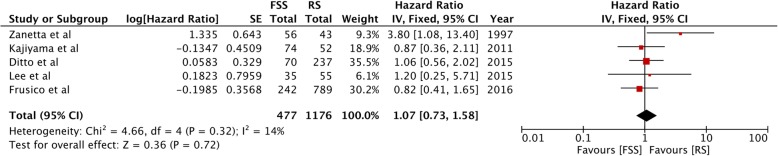
Forrest plot of hazard ratios for disease-free survival comparing fertility sparing surgery with radical surgery

**Fig. 5:**
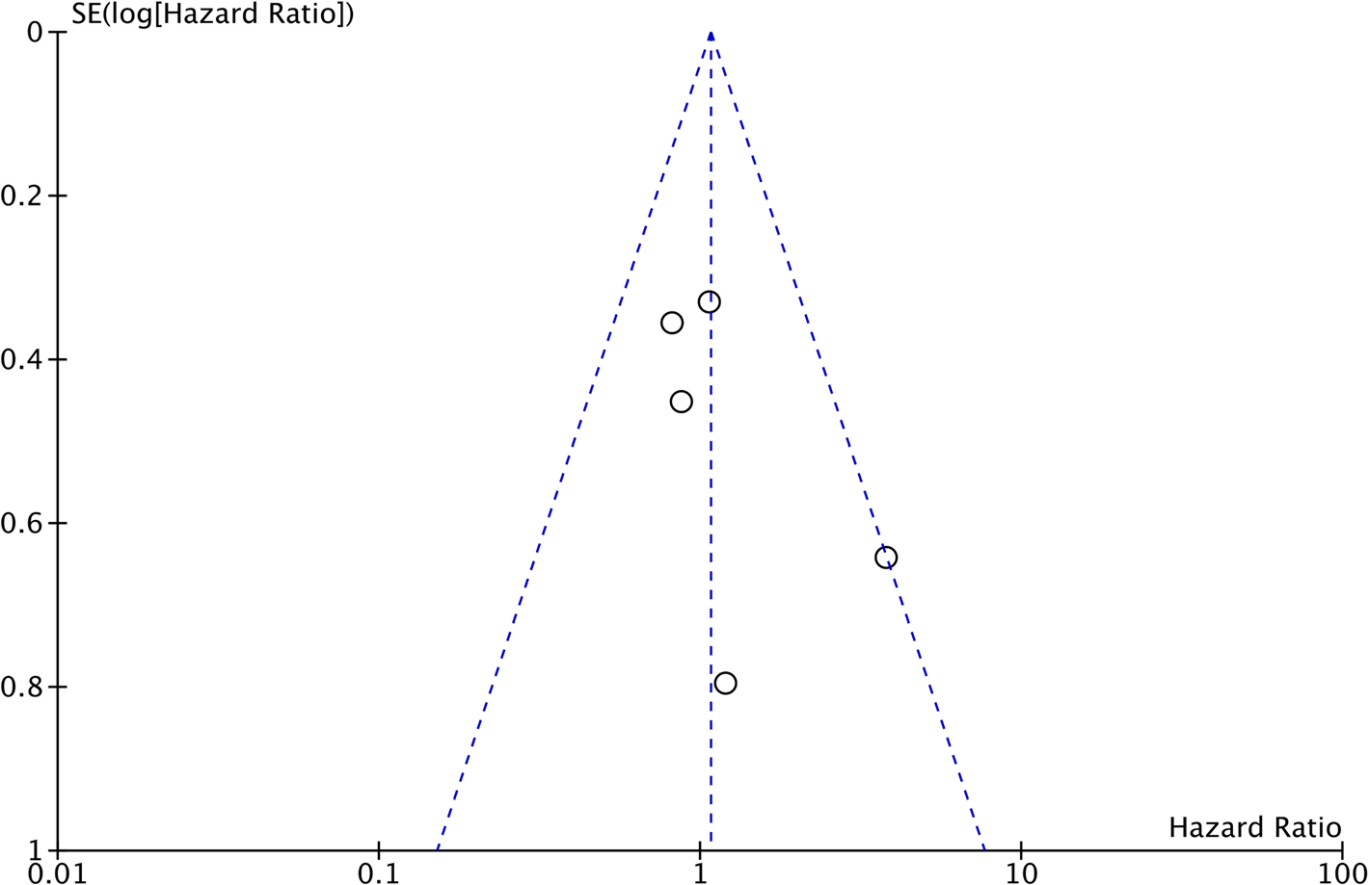
Funnel plot of studies comparing disease-free survival of fertility sparing surgery with radical surgery

Overall survival was reported from all eight studies. The pooled HR was non-significant (HR, 1.03; 95%CI, 0.80–1.31; *p* = 0.84) denoting no difference in overall survival between FSS and radical surgery (Fig. 2). There was no inter-study heterogeneity (I^2^ *= 0*%). Funnel plot indicated no evidence of publication bias (Fig. 3). Data on disease-free survival was available from five studies. Our analysis indicated no difference in disease-free survival between EOC patients undergoing FSS or radical surgery (HR, 1.07; 95%CI, 0.73–1.58; *p* = 0.72) (Fig. 4). Inter-study heterogeneity was minimal (I^2^ *=* 14%). No publication bias was evident on funnel plot (Fig. 5).

### Sensitivity analysis

Individual studies were sequentially excluded from the pooled effect to estimate the influence of each study on the overall outcome. The recalculated HRs on exclusion of each study for both overall survival and disease-free survival is presented in Table 2. There was no change in significance of HR on exclusion of any trial for both outcomes of interest.

**Table 2:** Results of sensitivity analysis

	Overall Survival		Recurrence free survival	
Excluded study	HR [95% CI]	*p*-value	HR [95% CI]	p-value
Crafton et al	0.96 [0.66, 1.39]	0.81	–	
Jiang et al	1.03 [0.81, 1.32]	0.79	–	
Frusico et al	1.03 [0.80, 1.33]	0.79	1.21 [0.76, 1.91]	0.43
Lee et al	1.03 [0.80, 1.32]	0.82	1.07 [0.72, 1.58]	0.75
Ditto et al	0.98 [0.76, 1.26]	0.86	1.08 [0.67, 1.74]	0.75
Kajiyama et al	1.04 [0.80, 1.34]	0.78	1.13 [0.73, 1.72]	0.59
Wright et al	1.12 [0.85, 1.48]	0.41	–	
Zanetta et al	0.99 [0.77, 1.27]	0.95	0.94 [0.63, 1.41]	0.77

HR, Hazard ratios; CI, Confidence interval

### Meta-regression

The proportion of patients with different EOC histology, sub-stage, grade and those receiving adjuvant radio/chemotherapy in the FSS group were calculated and analyzed for possible effect on pooled HR. Results of the meta-regression analysis for overall survival are presented in Table 3. None of the variables were found to have a statistically significant effect on HR. Meta-regression analysis for disease-free survival is presented in Table 4. Due to too few studies including clear cell tumors, meta-regression for proportion of clear-cell histology was not performed. Our results indicate that an increase in the proportion of patients with adjuvant therapy decreases HR, thus improving survival with FSS (*p* = 0.04).

**Table 3:** Meta-regression analysis for influence of dependent variables on overall survival

Variable	Coefficient	SE	−95% CI	+ 95% CI	p-value
Mucinous tumors	−0.0077	0.0098	−0.0269	0.0115	0.43
Endometroid tumors	−0.0076	0.0213	−0.0494	0.0341	0.72
Serous tumors	0.0186	0.0166	−0.0140	0.0511	0.26
Clear cell tumors	0.0065	0.0199	−0.0325	0.0455	0.74
Adjuvant Chemotherapy/radiotherapy	−0.0063	0.0033	−0.0127	0.0001	0.05
Stage 1A/1B tumors	−0.0067	0.0066	−0.0196	0.0062	0.30
Stage 1C tumors	0.0027	0.0100	−0.0169	0.0222	0.78
Grade 1 and 2	0.0102	0.0087	−0.0067	0.0272	0.23
Grade 3	0.0263	0.0275	−0.0276	0.0801	0.33

*SE* Standard Error, *CI* Confidence interval

**Table 4:** Meta-regression analysis for influence of dependent variables on disease free survival

Variable	Coefficient	SE	−95% CI	+ 95% CI	p-value
Mucinous tumors	−0.0019	0.0072	−0.0161	0.0123	0.79
Endometroid tumors	0.0024	0.0153	−0.0276	0.0324	0.87
Serous tumors	0.014	0.0101	−0.0058	0.0337	0.16
Adjuvant Chemotherapy/radiotherapy	−0.0141	0.0071	−0.028	−0.0001	0.04
Stage 1A/1B tumors	0.0153	0.0141	−0.0124	0.0429	0.27
Stage 1C tumors	−0.0056	0.0087	−0.0225	0.0114	0.51
Grade 1 and 2	0.0186	0.0227	−0.0259	0.0631	0.41
Grade 3	−0.0144	0.0197	−0.0531	0.0243	0.46

*SE* Standard Error, *CI* Confidence interval

## Discussion

For decades, the primary aim of managing early and advanced ovarian cancer has been to improve survival with little consideration to preservation of fertility. However, with evolving research and changing social trends, there has been increased emphasis on providing tailored treatment to young cancer patients desirous of childbearing in the near future. Especially in early EOC, with the disease confined to one ovary, gynecologists are increasingly proposing preservation of the uterus and unilateral ovary to women keen on retaining their childbearing ability [22]. This is in line with the American Society of Clinical Oncology (ASCO) updated guidelines of 2018 which recommend discussion of all fertility preservation options with ovarian cancer patients of childbearing age, one of which is FSS [23]. However, of utmost importance, is the safety of such conservative surgery so as not to reduce overall and disease-free survival.

Other than borderline, germ cell and stromal ovarian tumors, stage 1 EOC has also been a focus of research for FSS with several studies reporting good clinical outcomes [5, 14]. However, the level of evidence is either in the form of case series (Level 4) or at best from retrospective case-control studies (Level 3B) [24]. While it is recommended that clinical decisions should be based on evidence from the randomized controlled trials (RCTs) or systematic reviews and meta-analysis of RCTs, an RCT comparing FSS and radical surgery may not feasible due to technical and ethical issues. In the face of such dilemma, the primary aim of our study was to collate data from all case-control studies published till date so as to provide better evidence (if not the best) for FSS vs radical surgery for the management of early EOC.

On pooling of adjusted HR of more than 2000 patients undergoing FSS, our results indicate no significant difference in overall survival compared with radical surgery for stage 1 EOC. Our results concur with the growing body of literature which support FSS in early EOC [13, 21]. They also support the National Comprehensive Cancer Network guidelines that FSS may be carried out in select Stage 1 EOC patients where it is technically feasible [25]. It is important to note that the FSS was carried out in younger women in all included studies while radical surgery was predominantly carried out in older females. The impact of age on overall survival has been investigated by several studies [6, 20] and younger age has been associated with higher incidence of low grade tumors with better biological behavior [5]. Age was not adjusted in comparison of outcomes for the two surgical procedures in the included studies. However, data from propensity-score matched (including age-matched) cohorts has shown that the choice of surgical procedure does not influence outcomes [13]. Also, multivariate analysis have demonstrated that while increase in age has been independently associated with poorer prognosis, the choice of surgical procedure has no effect on overall survival [5].

To analyze the influence of tumor stage, histology, grade and use of adjuvant therapy on the overall HR, a meta-regression analysis was performed. Overall survival was not related to any disease stage, grade, histology or use of adjuvant therapy in our results. While there is greater consensus on the role of FSS for low-risk stage 1 EOC, some controversy still persists whether patients with stage 1 EOC with high risk features should undergo FSS [6]. Previous reports of poor survival in stage 1C disease, grade 3 tumors and patients with clear cell histology may have been influenced by the limited sample size studied [18, 26]. The results of our analysis concur with a propensity-scored matched analysis of Melamed et al. [13]. In a cohort of 904 patients, the authors reported no evidence of lower survival in patients with high risk features. The study of Crafton et al. [21] which is one of the largest retrospective review on this subject with an analysis of two cancer registries have also reported no influence of disease sub-stage, grade and histology on overall survival. To avoid overlap of data, details of only one cancer registry from this study were included in our analysis. However, since our analysis was restricted to Stage 1 EOC, conclusions cannot be drawn on the role of FSS for higher stage (>stage II) tumors which have been associated with poorer prognosis [21].

The second part of our meta-analysis was comparison of disease-free survival with FSS vs radical surgery. Pooled analysis demonstrated no difference in disease-free survival with FSS vs radical surgery in stage 1 EOC. However, data on recurrence was available only from five studies. Our results are similar to those reported by Bentivegna et al. [27] in their systematic review. On pooling of data from 32 studies, they reported a recurrence rate of 7% in stage IA grade 1 and 11% in stages IA grade 2 and stage IC grade 1/2 disease. These incidences were found to be similar to patients undergoing radical surgery. However, the recurrence rate for stage IC grade 3 was found to be higher than stage 1C grade 1 and 2. Disease-free survival can be influenced by baseline factors like tumor histology, tumor stage and tumor grade. To analyze such influence, a meta-regression was performed. Our results suggest that tumor histology (except clear-cell), tumor stage and tumor grade do not influence disease-free survival. However, we could not discern the influence of stage IC grade 3 tumors on the pooled HR for want of data. Previous small reviews on this subject have also reported similar results [28, 29]. Adjuvant therapy (mostly chemotherapy) was found to improve disease free-survival with FSS in our analysis, but the results were just statistically significant with the upper limit of CI just crossing zero value (− 0.0071 to − 0.0001).

The strengths of our study include a systematic literature search and pooling of HR rather than total number of events of death or recurrence. Pooling of number of events for a risk ratio or odds ratio would disregard the consideration of time in the analysis. Secondly, while pooling data from retrospective observational studies, it is important to adjust selection bias and potential confounders which can influence outcomes. We therefore abstracted adjusted risk estimates from included retrospective studies to conduct the current meta-analysis [30]. Meta-regression tool was used to analyze the influence of number of patients with specific baseline characteristic on the overall outcome.

The results of our study should be interpreted with the following limitations. Firstly, our study is a meta-analysis of observational studies only and at best provides level 3 evidence. The inherent bias of observational studies cannot be completely negated with a multivariate analysis and only a meta-analysis of robust homogenous RCTs can provide the highest level of evidence. Secondly, many of the included studies were retrospective analysis of cancer registries with no homogeneity in treatment selection, surgeon experience, central pathological review and type of adjuvant therapy. The comprehensiveness of the surgical procedure varied across studies. Thirdly, only eight studies were pooled for the meta-analysis with only three studies evaluating data of > 100 patients treated with FSS. Some studies also included stage II/III patients in the analysis. Although the number of such patients was < 10%, it may have influenced the overall results. Lastly, although funnel plots did not indicate any publication bias, power was limited due to the limited number of studies included in the analysis.

## Conclusions

To conclude, our study is the first systematic review and meta-analysis evaluating outcomes after FSS vs radical surgery for stage 1 EOC. On the basis of currently available observational studies there seems to be no difference in overall survival and disease-free survival with either surgical techniques for stage 1 EOC patients. Disease stage, tumor grade and histology does not appear to influence outcomes. Further homogenous studies shall improve the quality of evidence on this debatable subject.

## Supplementary information

**Additional file 1.** Search strategy and results of PubMed database.

## Data Availability

The datasets used and/or analyzed during the current study are available from the corresponding author on reasonable request.

## Abbreviations

FSS: fertility sparing surgery
EOC: epithelial ovarian cancer
CENTRAL: Cochrane Central Register of Controlled Trials
HR: hazard ratios
PRISMA: Preferred Reporting Items for Systematic Reviews and Meta-analyses
ASCO: American Society of Clinical Oncology
RCTs: randomized controlled trials
