# Supplementary material for: Fertility sparing surgery vs radical surgery for epithelial ovarian cancer: a meta-analysis of overall survival and disease-free survival
Source: BMC Cancer. 2020 Apr 15;20:320. doi: 10.1186/s12885-020-06828-y (PMC7161302; doi:10.1186/s12885-020-06828-y)
Supplement: Supplementary file 1 — Additional file 1. Search strategy and results of PubMed database. [file 12885_2020_6828_MOESM1_ESM.docx]

Additional file 1: Search strategy and results of PubMed database

| **Search number** | **Query** | **Search Details** | **Results** |
| --- | --- | --- | --- |
| **1** | (Fertility sparing surgery) AND (ovarian cancer) | ((((("fertiles"[All Fields] OR "fertility"[MeSH Terms]) OR "fertility"[All Fields]) OR "fertile"[All Fields]) OR "fertilities"[All Fields]) AND ((("spare"[All Fields] OR "spared"[All Fields]) OR "spares"[All Fields]) OR "sparing"[All Fields]) AND (((((((((("surgery"[MeSH Subheading] OR "surgery"[All Fields]) OR "surgical procedures, operative"[MeSH Terms]) OR (("surgical"[All Fields] AND "procedures"[All Fields]) AND "operative"[All Fields])) OR "operative surgical procedures"[All Fields]) OR "general surgery"[MeSH Terms]) OR ("general"[All Fields] AND "surgery"[All Fields])) OR "general surgery"[All Fields]) OR "surgery s"[All Fields]) OR "surgerys"[All Fields]) OR "surgeries"[All Fields])) AND (((("ovarian neoplasms"[MeSH Terms] OR ("ovarian"[All Fields] AND "neoplasms"[All Fields])) OR "ovarian neoplasms"[All Fields]) OR ("ovarian"[All Fields] AND "cancer"[All Fields])) OR "ovarian cancer"[All Fields]) | 545 |
| **2** | (Fertility sparing surgery) AND (epithelial ovarian cancer) | ((((("fertiles"[All Fields] OR "fertility"[MeSH Terms]) OR "fertility"[All Fields]) OR "fertile"[All Fields]) OR "fertilities"[All Fields]) AND ((("spare"[All Fields] OR "spared"[All Fields]) OR "spares"[All Fields]) OR "sparing"[All Fields]) AND (((((((((("surgery"[MeSH Subheading] OR "surgery"[All Fields]) OR "surgical procedures, operative"[MeSH Terms]) OR (("surgical"[All Fields] AND "procedures"[All Fields]) AND "operative"[All Fields])) OR "operative surgical procedures"[All Fields]) OR "general surgery"[MeSH Terms]) OR ("general"[All Fields] AND "surgery"[All Fields])) OR "general surgery"[All Fields]) OR "surgery s"[All Fields]) OR "surgerys"[All Fields]) OR "surgeries"[All Fields])) AND (((("carcinoma, ovarian epithelial"[MeSH Terms] OR (("carcinoma"[All Fields] AND "ovarian"[All Fields]) AND "epithelial"[All Fields])) OR "ovarian epithelial carcinoma"[All Fields]) OR (("epithelial"[All Fields] AND "ovarian"[All Fields]) AND "cancer"[All Fields])) OR "epithelial ovarian cancer"[All Fields]) | 119 |
| **3** | (Fertility sparing surgery) AND (epithelial ovarian tumours) | ((((("fertiles"[All Fields] OR "fertility"[MeSH Terms]) OR "fertility"[All Fields]) OR "fertile"[All Fields]) OR "fertilities"[All Fields]) AND ((("spare"[All Fields] OR "spared"[All Fields]) OR "spares"[All Fields]) OR "sparing"[All Fields]) AND (((((((((("surgery"[MeSH Subheading] OR "surgery"[All Fields]) OR "surgical procedures, operative"[MeSH Terms]) OR (("surgical"[All Fields] AND "procedures"[All Fields]) AND "operative"[All Fields])) OR "operative surgical procedures"[All Fields]) OR "general surgery"[MeSH Terms]) OR ("general"[All Fields] AND "surgery"[All Fields])) OR "general surgery"[All Fields]) OR "surgery s"[All Fields]) OR "surgerys"[All Fields]) OR "surgeries"[All Fields])) AND ((("epithelial"[All Fields] OR "epithelially"[All Fields]) OR "epithelials"[All Fields]) AND (((("ovarian neoplasms"[MeSH Terms] OR ("ovarian"[All Fields] AND "neoplasms"[All Fields])) OR "ovarian neoplasms"[All Fields]) OR ("ovarian"[All Fields] AND "tumours"[All Fields])) OR "ovarian tumours"[All Fields])) | 119 |
| **4** | (Fertility sparing surgery) AND (Conservative surgery) | ((((("fertiles"[All Fields] OR "fertility"[MeSH Terms]) OR "fertility"[All Fields]) OR "fertile"[All Fields]) OR "fertilities"[All Fields]) AND ((("spare"[All Fields] OR "spared"[All Fields]) OR "spares"[All Fields]) OR "sparing"[All Fields]) AND (((((((((("surgery"[MeSH Subheading] OR "surgery"[All Fields]) OR "surgical procedures, operative"[MeSH Terms]) OR (("surgical"[All Fields] AND "procedures"[All Fields]) AND "operative"[All Fields])) OR "operative surgical procedures"[All Fields]) OR "general surgery"[MeSH Terms]) OR ("general"[All Fields] AND "surgery"[All Fields])) OR "general surgery"[All Fields]) OR "surgery s"[All Fields]) OR "surgerys"[All Fields]) OR "surgeries"[All Fields])) AND ((((((((((((("conservancies"[All Fields] OR "conservancy"[All Fields]) OR "conservancy s"[All Fields]) OR "conservation"[All Fields]) OR "conservational"[All Fields]) OR "conservations"[All Fields]) OR "conservative"[All Fields]) OR "conservatively"[All Fields]) OR "conservatives"[All Fields]) OR "conserve"[All Fields]) OR "conserved"[All Fields]) OR "conserves"[All Fields]) OR "conserving"[All Fields]) AND (((((((((("surgery"[MeSH Subheading] OR "surgery"[All Fields]) OR "surgical procedures, operative"[MeSH Terms]) OR (("surgical"[All Fields] AND "procedures"[All Fields]) AND "operative"[All Fields])) OR "operative surgical procedures"[All Fields]) OR "general surgery"[MeSH Terms]) OR ("general"[All Fields] AND "surgery"[All Fields])) OR "general surgery"[All Fields]) OR "surgery s"[All Fields]) OR "surgerys"[All Fields]) OR "surgeries"[All Fields])) | 387 |
| **5** | (Conservative surgery) AND (ovarian cancer) | ((((((((((((("conservancies"[All Fields] OR "conservancy"[All Fields]) OR "conservancy s"[All Fields]) OR "conservation"[All Fields]) OR "conservational"[All Fields]) OR "conservations"[All Fields]) OR "conservative"[All Fields]) OR "conservatively"[All Fields]) OR "conservatives"[All Fields]) OR "conserve"[All Fields]) OR "conserved"[All Fields]) OR "conserves"[All Fields]) OR "conserving"[All Fields]) AND (((((((((("surgery"[MeSH Subheading] OR "surgery"[All Fields]) OR "surgical procedures, operative"[MeSH Terms]) OR (("surgical"[All Fields] AND "procedures"[All Fields]) AND "operative"[All Fields])) OR "operative surgical procedures"[All Fields]) OR "general surgery"[MeSH Terms]) OR ("general"[All Fields] AND "surgery"[All Fields])) OR "general surgery"[All Fields]) OR "surgery s"[All Fields]) OR "surgerys"[All Fields]) OR "surgeries"[All Fields])) AND (((("ovarian neoplasms"[MeSH Terms] OR ("ovarian"[All Fields] AND "neoplasms"[All Fields])) OR "ovarian neoplasms"[All Fields]) OR ("ovarian"[All Fields] AND "cancer"[All Fields])) OR "ovarian cancer"[All Fields]) | 1,385 |
| **6** | (Conservative surgery) AND (epithelial ovarian tumours) | ((((((((((((("conservancies"[All Fields] OR "conservancy"[All Fields]) OR "conservancy s"[All Fields]) OR "conservation"[All Fields]) OR "conservational"[All Fields]) OR "conservations"[All Fields]) OR "conservative"[All Fields]) OR "conservatively"[All Fields]) OR "conservatives"[All Fields]) OR "conserve"[All Fields]) OR "conserved"[All Fields]) OR "conserves"[All Fields]) OR "conserving"[All Fields]) AND (((((((((("surgery"[MeSH Subheading] OR "surgery"[All Fields]) OR "surgical procedures, operative"[MeSH Terms]) OR (("surgical"[All Fields] AND "procedures"[All Fields]) AND "operative"[All Fields])) OR "operative surgical procedures"[All Fields]) OR "general surgery"[MeSH Terms]) OR ("general"[All Fields] AND "surgery"[All Fields])) OR "general surgery"[All Fields]) OR "surgery s"[All Fields]) OR "surgerys"[All Fields]) OR "surgeries"[All Fields])) AND ((("epithelial"[All Fields] OR "epithelially"[All Fields]) OR "epithelials"[All Fields]) AND (((("ovarian neoplasms"[MeSH Terms] OR ("ovarian"[All Fields] AND "neoplasms"[All Fields])) OR "ovarian neoplasms"[All Fields]) OR ("ovarian"[All Fields] AND "tumours"[All Fields])) OR "ovarian tumours"[All Fields])) | 208 |
